# Gut-derived lipopolysaccharide remodels tumoral microenvironment and synergizes with PD-L1 checkpoint blockade via TLR4/MyD88/AKT/NF-κB pathway in pancreatic cancer

**DOI:** 10.1038/s41419-021-04293-4

**Published:** 2021-10-30

**Authors:** Hanlin Yin, Ning Pu, Qiangda Chen, Jicheng Zhang, Guochao Zhao, Xuefeng Xu, Dansong Wang, Tiantao Kuang, Dayong Jin, Wenhui Lou, Wenchuan Wu

**Affiliations:** 1 Department of General Surgery, Zhongshan Hospital, Fudan University, Shanghai, 200032 China; 2 Cancer Center, Zhongshan Hospital, Fudan University, Shanghai, 200032 China

**Keywords:** Cancer microenvironment, Cancer immunotherapy

## Abstract

Lipopolysaccharide (LPS) as an important inflammatory mediator activates the innate/adaptive immune system. The existence of LPS in pancreatic ductal adenocarcinoma (PDAC) has been reported, however, its biological function in PDAC remains unclear. Here, we demonstrated that circulating and tumoral LPS was significantly increased by intestinal leakage in the orthotopic murine PDAC model, and LPS administration promoted T cell infiltration but exhaustion paradoxically in the subcutaneous murine PDAC model. By bioinformatic analysis, Toll-like receptor 4 (TLR4), LPS receptor, was further found to enrich in immune tolerance signaling in PDAC tissues. Then, a significant positive correlation was found between TLR4 and programmed death ligand-1 (PD-L1) in clinical PDAC tissues, as well as serum LPS and tumoral PD-L1. Meanwhile, LPS stimulation in vitro and in vivo obviously upregulated tumor PD-L1 expression, and effectively promoted cancer cells resistance to T cell cytotoxicity. Mechanistically, the activation of TLR4/MyD88/AKT/NF-κB cascade was found to participate in LPS mediated PD-L1 transcription via binding to its promoter regions, which was enhanced by crosstalk between NF-κB and AKT pathways. Finally, PD-L1 blockade could significantly reverse LPS-induced immune escape, and synergized with LPS treatment. Taken together, LPS can remodel tumor microenvironment, and synergize with PD-L1 blockade to suppress tumor growth, which may be a promising comprehensive strategy for PDAC.

## Introduction

PDAC is a devastating disease that causes the fourth leading cancer-related death with the lowest 5-year survival rate (only 10%) compared with other cancers [1]. Due to the extremely malignant biological properties in PDAC, current antitumor therapeutic approaches, such as surgical resection, chemotherapy, and radiotherapy received limited benefit in PDAC patients. In recent years, PD-1/PD-L1-relevant immune checkpoint blockade (ICB) have shown promising efficacy in several cancers including melanoma and lung cancer [2, 3]. PD-L1 is an important negative immune molecular that participates in host immunity homeostasis, and tumor-associated PD-L1 promotes tumor immune escape by combining with PD-1 on tumor-infiltrating lymphocytes (TILs) and inducing their exhaustion and apoptosis [4]. PD-1/PD-L1 based ICB showed poor efficiency in PDAC in clinical trials [5–7]. Robust evidence showed that the potential immunological biomarkers to predict the response rate of PD-1/PD-L1 immunotherapy contained PD-L1 expression and intra-tumoral lymphocytes infiltrations [8]. PDAC has been documented as a nonimmunogenic tumor and low lymphocyte infiltration, which could be one of the explanations for its resistance to ICB. Hence, exploring new adjuvant therapy to change PDAC immune microenvironment and boosting the PD-1/PD-L1 based ICB therapeutic effect is promising in PDAC.

LPS is the out-membrane of gram-negative bacteria and can cause acute or chronic inflammation. LPS is the specific agonist to trigger the TLR4 signaling pathway in immune cells and “fuel” the innate immune activation to defense against bacterial infection [9, 10]. LPS stimulation could induce the secretion of IL-2, TNF-α, type I interferon by activating TLR4 in dendritic cells or macrophages, and induced anti-tumor T cell activation [11]. TLR4 is a transmembrane protein that is extensively expressed on immune cells including monocytes, macrophages, and dendritic cells [12–14], and non-immunocytes including platelets and epithelium [15–17]. TLR4 is also expressed in multiple cancer cells and its function was mainly centered on the proliferation and invasion, which indicated high TLR4 expression in tumor tissues predicted poor prognosis [18–20]. As the activator of TLR4, the existence of LPS has been widely confirmed in cancers from recent studies [21]. However, the role of LPS in PDAC was still unclear.

In this study, we identified that LPS existed in PDAC tissues and was associated with intestinal permeability. LPS could promote the anti-tumor effect in vivo but induce tumor infiltrated T cell exhaustion as well. Through bioinformatic analysis, a positive correlation was found between TLR4 and PD-L1 expression in PDAC tissues, which hinted that LPS might induce PD-L1 expression in PDAC. The correlation between serum LPS bioactivity and tumoral PD-L1 was further verified, and the mechanism of LPS inducing PD-L1 expression in PDAC was explored. Lastly, LPS and PD-L1 blockade combinate therapy showed great therapeutic value in the murine PDAC models. Taken together, these data help to illuminate a new mechanism of intestine-derived LPS remodels PDAC immune microenvironment and provide a new sight to boost PD-L1 blockade therapeutic efficiency in PDAC patients.

## Results

### Gut-derived LPS was detected in PDAC and influenced tumor microenvironment

**Fig. 1:**
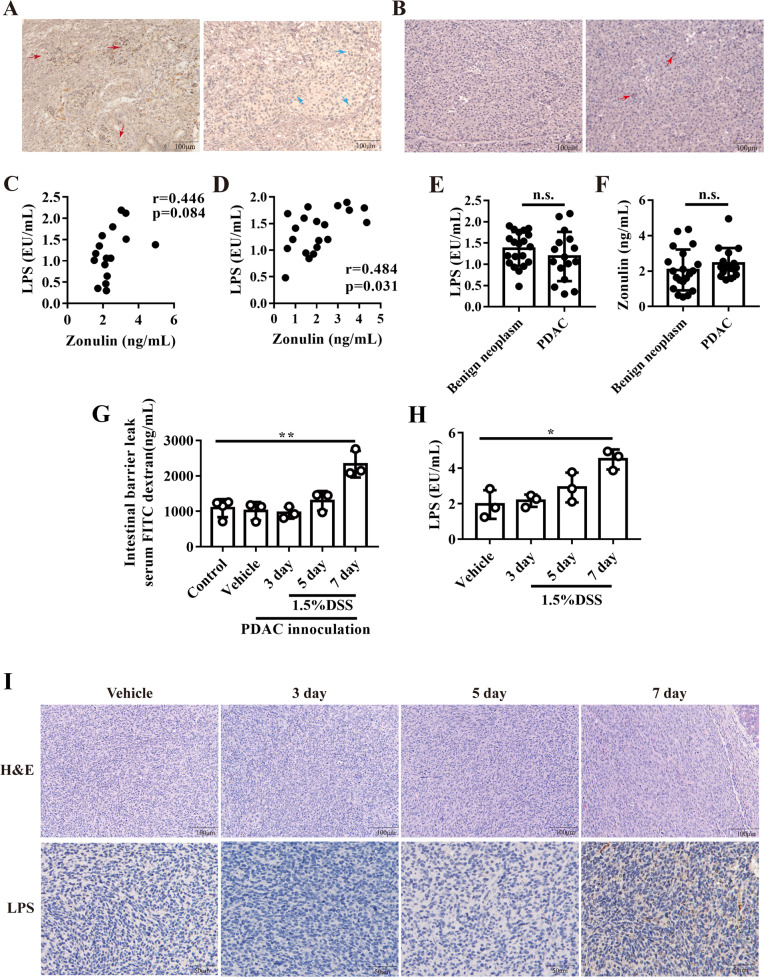
LPS existed in PDAC tissue and correlated with gut leakage. **A** the representative images of immunochemistry staining for LPS staining in pancreatic cancer tissues. **B** the representative images of immunochemistry staining for LPS staining in PDAC adjacent normal tissues. The correlation between serum LPS and Zonulin in PDAC patients (**C**) or benign pancreatic neoplasm patients (**D**). The serum LPS (**E**) and zonulin level (**F**) in patients with PDAC or benign pancreatic neoplasm. **G** FITC conjugated dextran were used to measure intestinal leakage after DSS treatment. **H** mice serum LPS were measured after DSS treatment. **I** the typical image of HE and LPS staining in orthotopic PDAC tissue.

Tumoral LPS was detected by immunochemistry staining as previously reported [21]. As expected, LPS could be detected in most of the human PDAC tissues (51/62) (Fig. 1A). In PDAC, LPS had strong positive staining in stroma and cytoplasm (red arrow in Fig. 1A), and weak positive staining in part of nuclei (blue arrow in Fig. 1A). LPS deposition also existed in adjacent normal tissues but was rare when compared to PDAC tissues (Fig. 1B). In healthy cases, serum circulating LPS was mainly derived from gut, which might explain the origins of LPS in tumor tissues. When intestinal permeability increased, LPS could transfer into host circulation and contribute to low-grade inflammation [22, 23]. One cohort containing 16 PDAC patients showed that the intestinal barrier marker - serum zonulin had a positive correlation with serum LPS in PDAC patients, which indicated that the increased intestinal leakage may cause a high level of serum LPS (Fig. 1C). Then, we enrolled 20 patients with pancreatic benign neoplasms as a control to detect LPS and zonulin levels. The results showed that LPS and zonulin had a positive correlation in patients with pancreatic benign neoplasms (Fig. 1D). In addition, the serum LPS (Fig. 1E) and zonulin level (Fig. 1F) showed no significant difference between patients with PDAC and benign neoplasms, which hinted that PDAC may not affect gut leakage. To confirm intestinal barrier-influenced serum and tumoral LPS, PDAC orthotopic murine models were established and DSS were used to destroy the gut barrier and cause gut leakage as previously reported (Figs. 1G and S1A) [24]. DSS treatment induced mice slight weight loss (Fig. S1B) and caused obviously colon colitis (Fig. S1C), colitis score showed that DSS treatment successfully induced murine colitis (Fig. S1D). Mice circulating LPS significantly increased after DSS treatment (Fig. 1H), as well as that in orthotopic tumor tissues after 7 days of DSS treatment (Fig. 1I). These results revealed that gut leakage might increase serum LPS and promote LPS deposition in PDAC tissues.

**Fig. 2:**
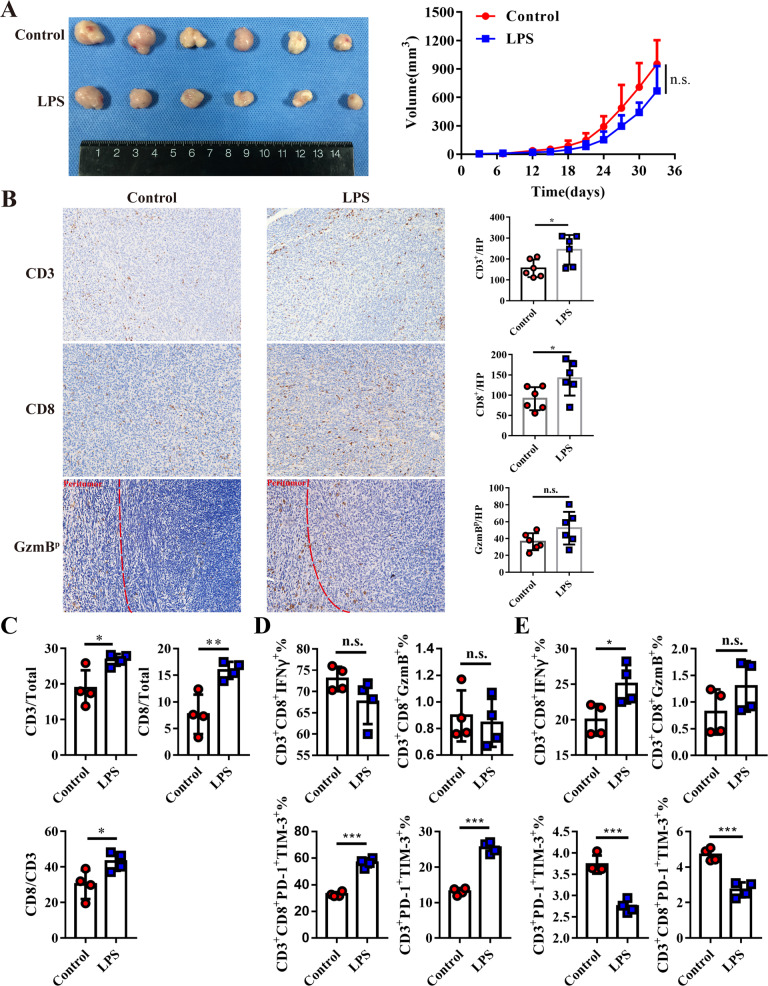
LPS induced systemic immune activation but also promoted TILs exhaustion in tumor tissue. **A** the photograph (left) and growth curve of subcutaneous tumor tissue were implanted into C57BL/6 and received LPS administration (100 µg/kg/3 days) or PBS as control (*n* = 6). **B** CD3, CD8, and GzmB were stained in the LPS and control groups. **C** flowcytometry analyzed the infiltration of TILs in murine Panc02 tumor tissue. GzmB, IFN-γ, PD-1, and TIM-3 phenotypes were used to evaluate the cytotoxicity or exhaustion of TILs (**D**) or spleen-derived T cells (**E**) by flow cytometry.

The effect of LPS in different cancers was controversial and was still unclear in PDAC. In order to explore the effect of LPS in PDAC, PDAC burden mice were regularly injected with LPS and their tumor growth rate was measured. Even though the tumor volume had no significant difference between the two groups (*p* = 0.084), a reduction trend in the LPS treatment group could be observed, which indicated that LPS could slightly inhibit tumor growth (Fig. 2A). As LPS is an immune activator, we speculated that LPS might change the tumor microenvironment and induce tumor shrink. Increased TILs including total CD3^+^ T cells and cytotoxic CD8^+^ T cells have been documented as prolonged survival index [25, 26], we analyzed TILs by immunochemistry staining and found that tumor-infiltrating CD3^+^ and CD8^+^ T cell significantly increased after LPS treatment (Fig. 2B). Granzyme B (GzmB) as secreted by CD8^+^ T lymphocytes and could cause cancer cell apoptosis. Peritumoral GzmB (GzmB^p^) was mildly increased after LPS treatment, however, no obviously statistical significance was observed (Fig. 2B). Tumor and spleen infiltrating CD3^+^ and CD8^+^ T cells, T-cell cytotoxicity makers GzmB^+^ and IFN-γ^+^, T-cell exhausted markers including TIM-3 and PD-1 were further identified by flow cytometry. The percentage of CD3^+^ T cell and CD3^+^CD8^+^ T in the total cell, the ratio of CD8/CD3 all obviously increased in tumor tissues after LPS treatment (Fig. 2C). The percentage of PD-1^+^TIM-3^+^ lymphocytes significantly increased after LPS treatment, but the percentage of IFNγ^+^ or GzmB^+^ lymphocytes showed no difference in tumor tissues, which revealed an increased exhausted tumor-infiltrating T cells after LPS treatment (Fig. 2D). However, the percentage of PD-1^+^TIM-3^+^ T cells were decreased, and IFNγ^+^ CD8^+^ T cell increased in spleen-derived T cells after LPS treatment (Fig. 2E). Considering the decreased exhaustion of peripheral T cells and increased exhaustion of intra-tumoral T cells, we speculated that LPS could activate systemic immune response, but induce T cell exhaustion in tumor tissues.

### TLR4 expression was higher in PDAC tumor tissues, which promoted immune activation and tolerance

**Fig. 3:**
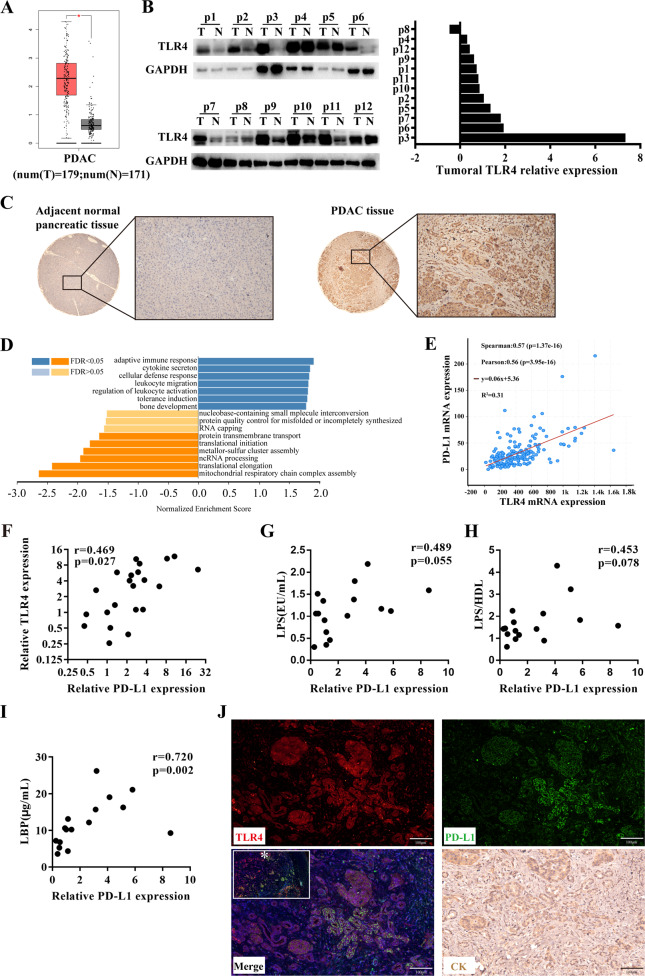
TLR4 was overexpressed in pancreatic cancer and induced both immune infiltration and immunosuppressive molecular PD-L1 in pancreatic cancer. **A** GEPIA analyzed TLR4 transcription data in PDAC tissues and normal tissues from the TCGA database. **B** western blot measured TLR4 protein expression in PDAC tumor tissues and adjacent normal tissues. **C** representative image of TLR4 expression in adjacent normal tissues and PDAC tissues. **D** LinkedOmics analyzed significantly enriched GO_BP annotations by GSEA of TLR4 in PDAC. **E** cBioportal tools analyzed the correlation between TLR4 and PD-L1 transcription in PDAC. **F** qRT-PCR measured TLR4 and PD-L1 mRNA expression in pancreatic cancer tissues. PDAC patient Serum LPS (**G**), serum LBP (**H**), and LPS/HDL (**I**) had a positive correlation with tumoral PD-L1 transcription. **J** representative image of TLR4 and PD-L1 co-immunofluorescence in PDAC tissues slice and CK19 staining in a continuous slice.

TLR4 is a crucial receptor that recognizes LPS and induces biological behavior to defense against bacteria. We first evaluated the expression of TLR4 and its role in PDAC specimens. Analysis of gene data about PDAC tissues and adjacent normal tissues originating from the TCGA database revealed that TLR4 mRNA was significantly higher in PDAC tissues compared to normal tissues (Fig. 3A). Then, TLR4 protein was further validated in 12 pairs of tumor tissues and adjacent normal tissues, eleven patients had higher expression of TLR4 protein in the tumor tissues (Fig. 3B). Immunochemistry staining further verified that TLR4 was overexpressed in PDAC cancer cells when compared to adjacent pancreatic ductal cells (Fig. 3C). These results suggested that TLR4 was overexpressed in PDAC tumor tissues and significantly associated with tumor progression.

In order to find the biological changes after LPS treatment in vivo, TLR4 co-expression genes were evaluated by LinkedOmics web-based platform. As shown in the volcano plot, the green curve and red curve separated the downregulated and upregulated genes (Fig. S2A). The top 50 genes positively (Fig. S2B left panel) and negatively (Fig. S2B right panel) correlated with TLR4 were shown in the heat maps. Furthermore, GSEA analysis indicated that TLR4 co-expressed genes mainly participated in immune activation, leukocyte migration, and tolerance induction (Fig. 3D), which might explain increased TILs, decreased exhaustion in the peripheral T cells, and increased exhaustion in tumor-infiltrating T cells. In addition, abnormal TILs exhaustion could explain no survival benefit from TLR4-related immune activation (Fig. S2C), which was consistent with previous reports [20].

In the analysis of immune tolerance-related genes, TLR4 was found to have a significant correlation with PD-L1 by analyzing 177 cases from the TCGA database (Fig. 3E), which was further validated in our own cohort (Fig. 3F). PD-L1 is a co-inhibitory molecule functioning as induction of T cell suppression and exhaustion, which may overcome TLR4-induced immune activation. To further explore the relationship between LPS and PD-L1 transcription in PDAC patients, another validation for the relationship between serum LPS level and paired tumoral PD-L1 expression in PDAC patients was implemented. A positive correlation was observed between serum LPS and PD-L1 (Fig. 3G). Similarly, LPS-binding protein (LBP) and LPS/high-density lipoprotein (HDL) were two main biomarkers for evaluating serum LPS activity as previously reported [27, 28]. As expected, further analyses showed that both LPS/HDL (Fig. 3H) and LBP (Fig. 3I) had a positive correlation with tumoral PD-L1. These showed that serum LPS had a positive correlation with tumoral PD-L1 transcription in PDAC patients. In addition, the relationships between TLR4 and other clinicopathological characteristics were displayed in Table 1, but no significant correlation was found. Then, co-immunofluorescence staining in PDAC tissues further showed that TLR4 and PD-L1 had co-expression in PDAC cells, which hinted at a potential correlation between these two proteins (Fig. 3J).

**Table 1:** The relationship between tumoral TLR4 expression and clinicopathological characteristics in resected PDAC patients.

Characteristics	Low TLR4 (*n* = 11)	High TLR4 (*n* = 11)	*P* value
***Gender***			
Male/Female	6/5	6/5	1
***Age***			
<70≥70	7/4	9/2	0.635
***Differentiation***			
Low/Moderate or High	6/5	5/6	1
***T classification***			
≤4 cm/>4 cm	2/9	3/8	1
***N classification***			
N0/N1-2	6/5	3/8	0.387
***TNM stage***			
I / II or III	5/6	2/9	0.361
***CA19-9***			
<37/≥37 U/L	5/6	5/6	1
***CEA***			
<5/≥5 ng/mL	9/2	4/7	0.08
***TBIL***			
≤20.4/>20.4 mmol/L	4//7	3/8	1
***Glucose***			
≤5.6/>5.6 mmol/L	6/5	6/5	1
***Albumin***			
<35/≥35 g/L	0/11	1/10	1

### LPS promoted immune escape via upregulating PD-L1

**Fig. 4:**
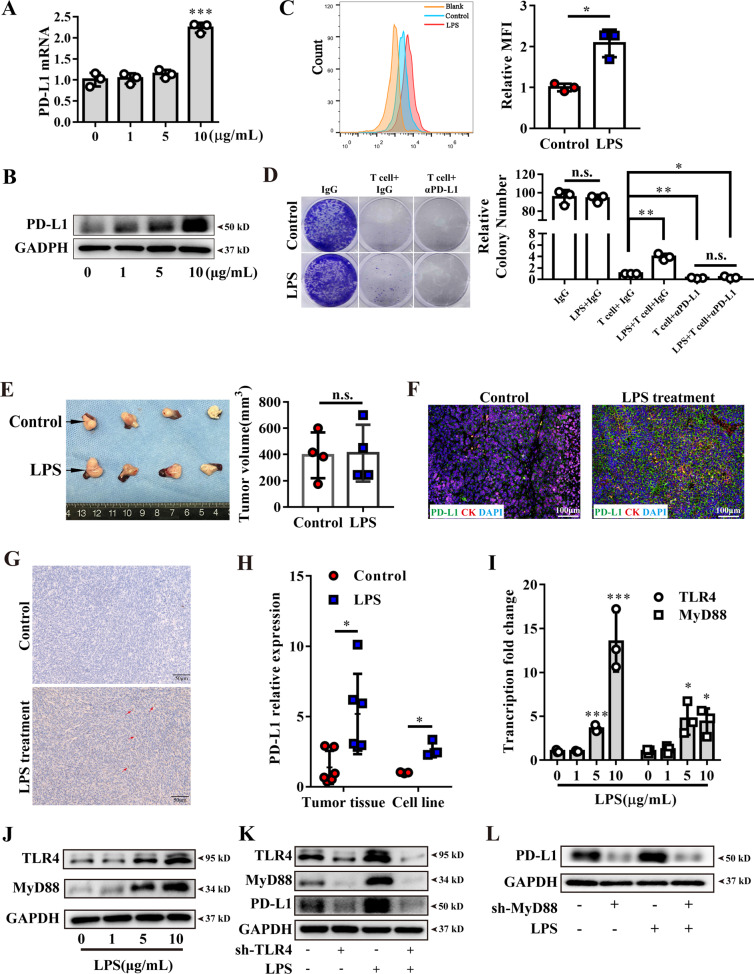
LPS induced PD-L1 expression in vitro and vivo and activated TLR4 signaling. **A** PD-L1 transcription were upregulated in PANC-1 after 10 ug/mL LPS stimulation for 6 h. Increased PD-L1 protein in whole-cell lysis (**B**) and cell surface (**C**) were identified by western blot or cytometry separately after LPS stimulation for 24 h. **D** T cell cytotoxicity to cancer cells were measured by PANC-1 clonal formation assay. Cancer cells were pre-stimulated by 10 μg/mL LPS or PBS as a control for 24 h and T cells were seeded into the well. Anti-PD-L1 antibody (αPD-L1) were used to blockade PANC-1 endogenous PD-L1, and isotype IgG was used as control. **E** the photograph and tumor volume of PANC-1 orthotopic xenograft tumor in the end of the experiment (*n* = 4). **F** PD-L1 (green) and CK19 (red) co-immunofluorescence staining were analyzed for PD-L1 expression in PANC-1 xenografts after LPS treatment. **G** the representative PD-L1 staining of Panc02 derived subcutaneous tumors. **H** qRT-PCR showed PD-L1 transcription level in Panc02 derived tumor tissue with or without LPS administration in vivo and 10 μg/mL LPS stimulation for 6 h in Panc02 cell lines in vitro. qRT-PCR assay (**I**) and western blot (**J**) showed TLR4 and MyD88 expression after 10 μg/mL LPS stimulation for 24 h in PANC-1. **K** western blot analyzed TLR4, MyD88, and PD-L1 expression after TLR4 knockdown in PANC-1. **L** western blot showed that sh3-MyD88 knockdown decreased PD-L1 and restrained LPS effect in PANC-1. *, **, *** and n.s. means statistically significant difference at *p* < 0.05, *p* < 0.01, *p* < 0.001 and no significant, respectively.

To further confirm LPS inducing PD-L1 expression in PDAC, we firstly detected TLR4 and PD-L1 basal expression in four pancreatic cancer cell lines and normal pancreatic epithelia cell line (Fig. S3A). We chose PANC-1 and BxPC-3 for further study, as PANC-1 had the lowest TLR4 expression, while BxPC-3 had the highest TLR4 expression. Then, the cells were stimulated by varying LPS concentrations (0, 1, 5, 10 μg/ml). After stimulation, PD-L1 were up-regulated in PANC-1 (Fig. 4A–C) and BxPC-3 (Fig. S3B–D). Besides, no difference was found in the stimulated HPDE cells (Fig. S3E, F). T cell cytotoxicity assay was performed and found that T cell cytotoxicity to cancer cell was weakened after LPS pretreatment (Figs. 4D and S3G).

To further verify the effect of LPS on inducing PD-L1 in vivo, orthotopic PDAC murine models were established. Orthotopic pancreatic cancer tissues were harvested to analyze after being administered with LPS for 5 doses (Figs. 4E and S3H), and no difference of tumor volumes between these two groups was observed. However, tumor tissues in the LPS-treated group had obviously higher PD-L1 expression than the control group (Figs. 4F and S3I). These results may partly result from limited doses of LPS treatment, but obviously revealed that LPS could promote PD-L1 expression in PDAC.

PD-L1 were both upregulated in the Panc02 cell lines, or its subcutaneous tumor tissues after LPS treatment (Fig. 4G, H), which could explain the increased exhaustion in the tumor-infiltrating T cells. Thus, these results revealed that LPS was one of the crucial factors promoting tumor PD-L1 transcription in pancreatic cancer cells.

### LPS upregulated PD-L1 though TLR4/MyD88/AKT/NF-κB signaling pathway

TLR4 is known as the important receptor for recognizing LPS, and then its downstream molecular, MyD88, is recruited for initiating TLR4-related cascade [29, 30]. TLR4 and MyD88 were found to be significantly increased in a concentration-dependent manner in PANC-1 (Fig. 4I, J) and BxPC-3 (Fig. S4A, B), which hinted that stimulating TLR4 led to positive feedback in pancreatic cancer cells. To further verify the mechanism, TLR4-specific sh-RNA plasmid was used to knockdown endogenous TLR4, and the TLR4-sh2 plasmid was chosen for the following studies (Fig. S4C). The result showed that PD-L1 and MyD88 expression was significantly decreased when TLR4 was knocked down (Figs. 4K and S4D). MyD88 sh-plasmid was also used to intervene endogenous MyD88, and MyD88-sh3 showed the best performance (Fig. S4E). When MyD88 was knocked down, PD-L1 was proved to be significantly decreased (Figs. 4L and S4F). These results confirmed that TLR4/MyD88 participated in LPS-induced PD-L1 expression.

**Fig. 5:**
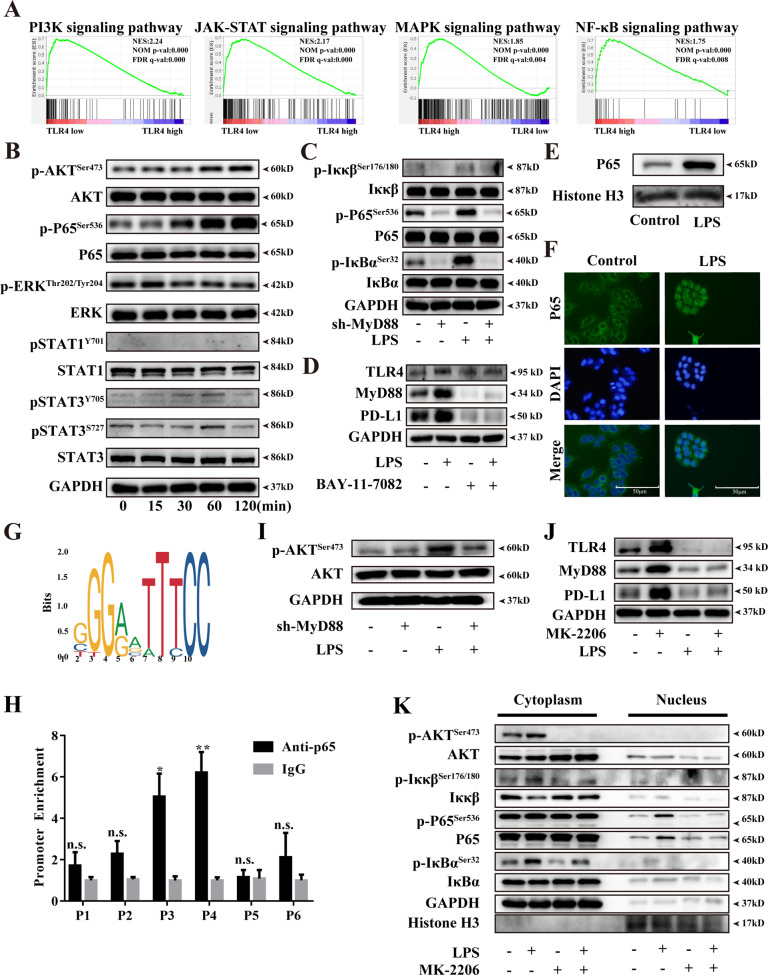
LPS induced PD-L1 expression was MyD88-dependent/AKT/NF-κB pathway. **A** GSEA plots of JAK-STAT, NF-κB, PI3K, and MAPK pathway showing a positive correlation with higher TLR4 in PDAC. FDR, false-discovery rate *q* value. NES, normalized enrichment score. **B** NF-κB, AKT, MAPK, and JAK-STAT3 pathway phosphorylation was measured by western blot after LPS stimulation in a time-dependent manner. **C** NF-κB pathway activation was measured by western blot after Sh3-MyD88 knockdown in PANC-1. **D** TLR4/MyD88/PD-L1 expression were measured after NF-κB inhibitor Bay-11-7082 (10 μM) pretreatment in PANC-1. **E** western blot measured P65 nucleus translocation in PANC-1. **F** immunofluorescent staining analyzed P65 translocation after LPS stimulation in PANC-1. 10 μg/mL LPS was used to stimulate cells for 1 h and P65 was detected by using Alexa Fluor 488 conjugated goat anti-rabbit IgG. **G** JASPAR program showed the seed binding sites of the P65 transcription factor. **H** CHIP assay showed the combination of P65 subunit and PD-L1 promoter in PANC-1. Rabbit IgG were used as a negative control. **I** AKT pathway activation was measured by western blot after sh3-MyD88 knockdown in PANC-1. **J** PD-L1 expression was detected by western blot after AKT pathway inhibitor, MK-2206, pretreatment for 2 h in PANC-1. **K** western blot showed that MK-2206 pretreatment inhibited LPS mediated P65 translocation without changing IκBα phosphorylation. *, ** and n.s. means statistically significant difference at *p* < 0.05, *p* < 0.01 and no significant.

To further illustrate the potential downstream of TLR4 in PDAC, 178 PDAC cases from the TCGA database were divided into two groups ranked by their TLR4 expression. GSEA was used for identifying gene signatures that had a close correlation with TLR4. Four potential signaling pathways (NF-κB pathway, PI3K pathway, JAK-STAT pathway, and MAPK pathway) were significantly enriched in high TLR4 expressed tissues, which might be downstream of TLR4 and participate in PD-L1 expression in PDAC (Fig. 5A). We further detected whether these four signaling pathways were activated after LPS stimulation. As a result, the canonical MAPK pathway had no obvious change after LPS treatment, which indicated that the MAPK pathway didn’t involve in LPS-induced PD-L1 transcription in PDAC cell lines. Phos-STAT1 could not be detected in cell lysate, and no obvious time-dependent phos-STAT3 changes after LPS stimulation were found. AKT and NF-κB pathways were both steadily activated after stimulation (Figs. 5B and S5A). Based on these findings, we speculated that AKT and NF-κB mainly participated in LPS-induced PD-L1 transcription in PDAC. To explore whether LPS induced AKT and NF-κB activation is MyD88-dependent or not, these two signaling pathways were further detected after MyD88 was knocked down. The results showed that NF-κB phosphorylation was decreased after MyD88 knockdown and cannot be rescued by LPS stimulation (Figs. 5C and S5B). The optimal concentration of IκBα phosphorylation inhibitor was determined in two PDAC cell lines (Fig. S5C). The pretreatment of the NF-κB inhibitor obviously decreased the expression of PD-L1, which indicated that the NF-κB pathway participated in PD-L1 transcription in PDAC cancer cells (Figs. 5D and S5D). NF-κB is a nuclear transcriptional factor that consists of a P65-p50 heterodimer. When phosphorylated in the cytoplasm by IκB kinase, the P65 subunit transfers to the nucleus and binds to a specific promoter, and starts gene transcription [31]. To clarify P65 participated in PD-L1 transcription after LPS stimulation, cytoplasm and nucleus proteins from cancer cells treated LPS stimulation or PBS were separated to measure P65 translocation (Figs. S5E). Western blot (Fig. 5E and S5F) and immunofluorescence (Figs. 5F and S5G) both revealed that LPS treatment could obviously promote P65 nuclear translocation. Concurrently, the potential binding sites of PD-L1 were predicted by JASPAR (Fig. 5G), and 6 potential binding sites were further detected by CHIP-qPCR assays. The results showed that the P65 subunit could combine with the PD-L1 gene promoter, and had obvious enrichment compared to IgG control (Figs. 5H and S5H). These data demonstrated that P65 could directly occupy the promoter regions of PD-L1 in PDAC after LPS stimulation.

It is widely accepted that the NF-κB pathway is the crucial downstream of the TLR4 signaling pathway [18, 19], AKT pathway was also activated after LPS stimulation. In order to explore whether AKT activation is MyD88-dependent or not, MyD88 was knockdown as previously and found that AKT pathway phosphorylation also decreased (Figs. 5I and S6A). This indicated that AKT pathway activation in LPS stimulation was also in a MyD88-dependent way. The optimal AKT pathway inhibitor concentration was selected (Fig. S6B) and its pretreatment also reduced PD-L1 expression (Figs. 5J and S6C). This demonstrated that the AKT pathway also participated in LPS-induced PD-L1. To explore the potential interaction in NF-κB and AKT pathway, cancer cells were pretreated with bay-11-7082 and found that AKT phosphorylation obviously decreased after IκBα inhibition (Figs. S6D). This hinted that increased IκBα phosphorylation could lead to AKT pathway activation. In turn, after pretreatment with AKT inhibitor, the phosphorylation of NF-κB didn’t change, which seemed that the AKT pathway didn’t affect LPS mediated NF-κB activation (Figs. S6E). To explore whether AKT pathway inhibition affected P65 translocation, we measured cytoplasmic/nuclear cell lysate after AKT phosphorylation inhibition and found that P65 translocation obviously reduced even though IκBα phosphorylation didn’t change (Figs. 5K and S6F). This showed that AKT phosphorylation could assist P65 translocation independent of intervening IκBα-P65 dissociation. From the above results, LPS stimulated NF-κB pathway transcription included direct NF-κB activation and AKT-mediated indirect mechanism.

### LPS treatment synergized with PD-L1 blockade responses in PDAC murine models

**Fig. 6:**
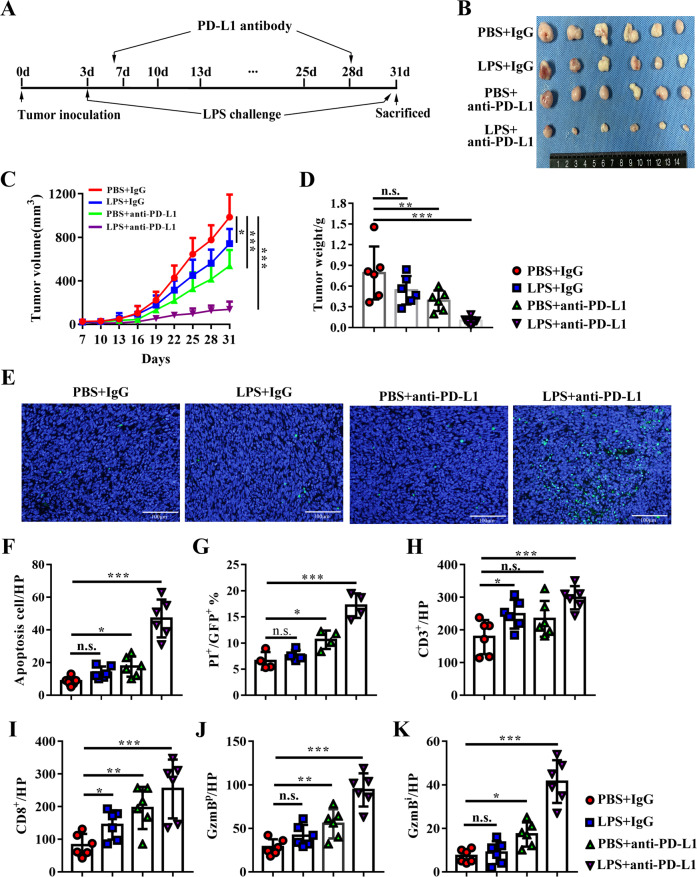
LPS enhanced PD-L1 blocking therapy in PDAC murine model by promoting anti-tumor immune. **A** the process of LPS and anti-PD-L1 therapy animal experiment. **B** subcutaneous tumor derived from PBS + IgG isotype group, LPS + IgG isotype group, PBS + anti-PD-L1 group, and LPS + anti-PD-L1 group at the end of the experiments (*n* = 6). Tumor growth curve (**C**) and tumor volume (**D**) of tumor derived from each group. **E** representative image of tunnel staining in each group to evaluate apoptotic cells. **F** apoptosis cell was calculated as tunnel^+^ cells/ visual filed. **G** apoptotic cancer cells in each group was evaluated by calculating the percentage of PI-positive cells in all GFP-positive cells (*n* = 4). Tumor infiltrated CD3^+^ (**H**) and CD8^+^ (**I**) T cells were stained by immunochemistry and analyzed as positive staining number per high visual field (HP). Peritumoral GzmB (**J**) and intratumoral GzmB (**K**) distribution were calculated as GzmB^p^/percentage of the peritumoral area or GzmB^i^/percentage of peritumoral area in a visual field, separately. *, **, *** and n.s. means statistically significant difference at *p* < 0.05, *p* < 0.01, *p* < 0.001 and no significant, respectively.

As we have proved that LPS could inhibit tumor growth, but induce PD-L1 expression in PDAC cells, we speculated that anti-PD-L1 immunotherapy might had a synergistic effect with high systemic LPS on PDAC burden mice. We established a subcutaneous PDAC murine model and treated it with LPS or/and PD-L1 blockade antibody (Fig. 6A). As expected, PD-L1 monotherapy could both reduce tumor volume. LPS monotherapy significantly reduced tumor volume in this study (*p* = 0.036) but tumor weight didn’t have a significant difference, this was because of the irregular shape of the tumor cannot get accuracy volume when measured only by the major and minor axis of tumor maximum cross-section. However, LPS could boosted the efficacy of PD-L1 blocking therapy (Fig. 6B–D). Considering PD-L1 was an immunosuppressive molecular and induced TILs exhausted and apoptosis, tumor infiltration lymphocytes, and their cytotoxicity were examined. The apoptotic cells in tumor tissues were also synergistically increased when compared to monotherapy (Fig. 6E, F). To further evaluate cancer cell apoptosis precisely, the GFP stable expressed Panc02 cell lines were established and repeated in the same experiment. The percentage of PI^+^GFP^+^ cells in all GFP^+^ cells was calculated, and the results were in consistent with the tunnel staining (Fig. 6G). The infiltration of CD3^+^, CD8^+^ T lymphocytes were significantly increased after monotherapy, and combination therapy promoted more TILs in tumor tissues (Figs. 6H, I and S7). GzmB^p^ was also notably increased after PD-L1 was blocked when comparing with the IgG control group, and combination therapy further increased GzmB^p^ than monotherapy (Fig. 6J). Intra-tumoral GzmB (GzmB^i^) was rare in both PBS and LPS groups, even though there remains significant CD8^+^ T lymphocyte infiltration. This hinted potential intense immunosuppression existed in the intra-tumoral microenvironment and PD-L1 monotherapy increased GzmB^i^ (Fig. 6K), which indicated that intra-tumoral immunosuppression may be partly reversed by PD-L1 blockade. Therefore, these results supported that systemic low-grade LPS could effectively synergize with PD-L1 immunotherapy for PDAC.

## Discussion

**Fig. 7:**
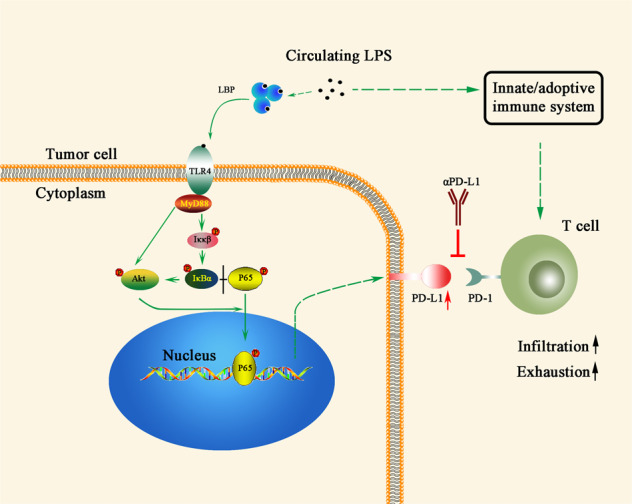
The schematic drawing of LPS function in PDAC. Gut-derived LPS is double-edged sword to PDAC, which induced T cell infiltration in tumor microenvironment and promoted PD-L1-mediated immune evasion as well. Resultantly, LPS had synergistic effect with PD-L1 blockade in PDAC.

The intestinal barrier is linked by epithelia cells and their tight junction proteins that promote nutrient absorption and prevent translocation of intraluminal bacteria and their products. Abundant evidence showed the closely link between the gut-intestinal microbiome and cancer. Here we found a new mechanism that intestinal leakage increased systemic circulation LPS and it further induced intra-tumoral lymphocyte infiltration and as well as upregulated PD-L1 expression via TLR4 signaling pathway, which could promote tumor immune escape (Fig. 7).

LPS is an important PAMP to induce immune maturation and activation. Generally, it had been widely considered that body tissue, including tumor tissue, is germ-free. However, recent studies found the existence of LPS and bacterial DNA in tumor tissue [21, 32]. Coinciding with their results, our data gave a further validation of the existence of LPS in pancreatic cancer tissue. Gut microbiome translocation is one of the reasons to explain the existence of PDAC intra-tumoral LPS. Our finding put forward another potential mechanism of intra-tumoral LPS: intestinal barrier disruption induced high circulating LPS and increased LPS deposition in tumor tissue.

TLR4-deficient mouse intriguingly showed pro-tumor effect in skin, lung, and mammary cancers [33–35], which determined that loss of TLR4 signaling pathway impaired anti-tumor immunity. However, tumor-intrinsic TLR4 is negatively correlated with survival [20]. Our research showed a positive correlation between TLR4 and PD-L1 in the public database and further verified their correlation in our cohort. This could explain high intra-tumoral TLR4 expression indicating a worse prognosis. In our study, LPS monotherapy had a weak therapeutic effect and could not get prominent efficacy in reducing tumor growth, so LPS monotherapy may not be a good choice for PDAC treatment. TILs are relatively rare in PDAC when comparing with “immune inflamed” cancers like melanoma, which is the limitation that causes disappointing outcomes in PDAC immunotherapy. LPS could obviously increase tumor infiltrated lymphocytes in our study, this hinted that LPS had potential value used as an immunological adjuvant in ICB therapy in pancreatic cancer.

As for PDAC patient, in our cohort none of the patients had infectious evidence when collecting samples, this hinted that LPS could be absorbed from the gastrointestinal tract. The two steps of LPS activating TLR4/MD2 complex includes LPS binding to LBP [27], and LBP delivers LPS to CD14 which then interacts with TLR4 [36]. As LBP helped the formation of the LPS/TLR4 complex and enhanced the host sensitivity of LPS, LBP was suggested as “effective” LPS [37]. It has been reported that LBP reaches to a peak rapidly after endotoxemia and maintains for at least 72 h [38]. So, LBP can reflect the long-term effect of LPS-related inflammation and had a better correlation with tumoral PD-L1 comparing with LPS itself.

In PDAC immunotherapy clinical trial PD-1/PD-L1 had a limited response rate [5]. According to our findings, a high level of serum LPS activity meant activated immune response, higher tumoral infiltrated lymphocyte, but also elevated tumoral PD-L1 mediated immunosuppression. Higher level of immune response and PD-L1 expression are the important biomarker to predict PD-L1 blockade therapy response [8]. LPS also had been used as an immunotherapy adjuvant in melanoma without obvious side effect [39]. Our data indicated that circulating LPS could be regard as PD-1/PD-L1 ICB response rate predictive factor in PDAC and has great potential in boost PD-1/PD-L1 ICB efficacy as an immunologic adjuvant.

## Materials and methods

### Sample collections

All tumor tissues, matched adjacent tissues and pre-operational serum were prospectively collected from registered patients in Zhongshan Hospital, Fudan University from September 3, 2019 to December 3, 2019. Tumor tissues pathologically diagnosed as PDAC by experienced pathologists were recruited in our study. Enrollment criteria included: no anti-cancer treatment before operation; no evidence of preoperative infection; no history of other type of malignance; available clinicopathological data. The clinicopathological data of total in enrolled patients, including age, sex, differentiation grade, total bilirubin, albumin, CEA, CA19-9, TNM stage, glucose, were listed in Table S1. Another cohort including 20 patients with benign pancreatic neoplasms (serous cystic neoplasms, intraductal papillary mucinous neoplasm without atypical hyperplasia) were enrolled as the control. Study approval was obtained from the Clinical Research Ethic Committee of Zhongshan Hospital, Fudan University, and informed consents were received from all enrolled patients.

### Serum LPS, LBP and Zonulin measurements

Serum LPS was measured using a quantitative limulus kit (Xiamen Bioendo Technology, Fujian, China). Serum was diluted to 1:10 by LPS-free water and incubated with the reagents according to the manufacturer’s protocol. Serum LPS-binding protein (LBP) concentration was detected by LBP enzyme-linked immunosorbent assay kit (Boster Biological Technology Co., Ltd), the procedures were followed the manual from the manufacturer. Serum Zonulin concentration was measured by Zonulin enzyme-linked immunosorbent assay (Cusabio, Wuhan, China).

### Flow cytometry analysis

Tumor tissues was digested with collagenase I and IV (Gibco) and then ground by Tumor Dissociation Kit (Miltenyi Biotec) through gentleMACS (Miltenyi Biotec) to generate a single-cell suspension. The suspension was then filtered through 70μm filters. Cells washed twice by phosphate-buffered saline (PBS, pH 7.4) containing 2% fetal calf serum (FBS) (Gibco), stained with appropriate antibodies for 30 min, and detected by flow cytometry (Arial III, Becton-Dickinson Biosciences). As for intracellular staining, cells were pretreated with IC Fixation and Permeabilization Buffer Kit (Thermo fisher) and stained with antibodies.

### Functional enrichment analysis via gene set enrichment analysis (GSEA)

To investigate the related signaling pathway of TLR4 in PDAC, PDAC samples from the TCGA database were divided into two groups based on the TLR4 expression. Differential gene expression when comparing TLR4 high group to TLR4 low group were ranked based their testing statistics and the enrichment of gene sets were analyzed by GSEA. Significant enriched gene sets were identified as normal *P* value < 0.01, and false discovery rate (FDR) < 0.05.

### GEPIA database analysis

Gene Expression Profiling Interactive Analysis (GEPIA) web-based platform (http://gepia.cancer-pku.cn/) was used to analyze the gene expression profiling TCGA database [40]. TLR4 transcription profile and survival curve (Disease-Free Survival and Overall Survival) were generated by GEPIA.

### LinkedOmics database analysis

LinkedOmics database (http://linkedomics.org/login.php) is a publicly online analysis tools that includes multi-omics data from all 32 TCGA Cancer types [41]. The Gene Ontology biological process (GO_BP) of co-expressed genes were analyzed by GSEA. The rank criterion was FDR < 0.05.

### cBioportal database analysis

The cBio Cancer Genomics Portal (http://cbioportal.org) has multidimensional cancer genomics online platform [42]. TLR4 and PD-L1 transcriptional correlation was analyzed by cBioportal tool.

### Cell lines and animal model

The human pancreatic tumor cell lines MIA-PaCa-2, PANC-1, BxPC-3, Sw1990, and normal pancreatic epithelial cell line HPDE were obtained from Chinese Academic of Science and kept in our laboratory as previously reported [43]. Cells were authenticated and tested for mycoplasma contamination before the study. C57BL/6-derived pancreatic cancer cell line Panc02 was cultured in RPMI1640 with 10%FBS. Cells were incubated at 37 °C in 5% CO_2_ and 95% air humidified atmosphere.

Six-week-old female C57BL/6 mice (Shanghai Jie Si Jie Laboratory Animal Co., Ltd, Shanghai, China) were subcutaneously or orthotopically inoculated with 100 μL or 50 μL Panc02 cell suspension (2 × 10^7^/mL) mixed with Matrigel as 2:1. For DSS experiments, tumor burden mice start to be treated with 1.5% weight/volume DSS (MP Biomedicals, molecular weight 36000-50000) for 3, 5, 7 days on 20^th^ day and followed by regular drinking water until sacrificed. Histopathology activity index scores were evaluated as previously reported [44] and scoring system was listed in Table S2. LPS (Sigma Aldrich, USA) (100 μg/kg/day) were intraperitoneally injected three days after tumor inoculation per two days. PD-L1 antibody (Bio X cell) was intraperitoneal injected as 100 μg/mouse per three days.

As for cell line derived xenograft model, six-week-old male BALB/c background nude mice (Shanghai Jie Si Jie Laboratory Animal Co., Ltd) were anesthetized by pentobarbital intraperitoneal injection. 50 μL PANC-1 or BxPC-3 cells suspension (4 × 10^7^/mL) mixed with Matrigel were injected into mice pancreas tail to establish orthotopic PDAC murine model. 100 μg/kg/day LPS or PBS as control were administrated peritoneally every day in the last five days. In the 25^th^ day, all the samples were excised immediately.

The animal was randomly allocated in a different groups by using the random number method with no blinding. Animal management complied with our Institutional Animal Care and Use Committee at Zhongshan Hospital, Fudan University, Shanghai, China.

### RNA extraction and quantitative real-time polymerase chain reaction (qRT-PCR)

The total RNAs of cells and tissues were isolated by TRIzol Reagent (Invitrogen, Carlsbad, CA, USA) and then applied for qRT-PCR by reverse transcriptase master mix and SYBR Premix Ex Taq (Takara Bio Inc., Otsu, Shiga, Japan) as our previous report [43]. The sequences of primers used for qRT-PCR were as follows: human TLR4,5-TGTGCAACACCTTCAGATAAGCA-3 (forward) and 5-ACAACAGATACTACAAGCACAC-3 (reverse); human MyD88, 5-CTGGCTGCTCTCAACATGCG-3 (forward) and 5-CCAGTTGCCGGATCTCCA-3 (reverse); human PD-L1, 5-CCAGG ATGGTTCTTAGACTCCC-3 (forward) and 5-TTTAGCACGAAGCTCTCCGAT-3 (reverse); human GAPDH, 5-CAACAGCCTCA AGATCATCAGC-3 (forward) and 5-ATGAGTCCTTCCACGATACCAA-3 (reverse); mouse PD-L1, 5-AGTCTCCTCGCCTGCAGATA-3 (forward) and 5-AGTAAACGCCCGTAGCAAGT-3 (reverse); mouse GAPDH, 5-GCCGAGAATGGGAAGCTTGTC-3 (forward) and 5-TCCACGACATACTCAGCACCG-3 (reverse). The fold change of TLR4, MyD88, and PD-L1 mRNA was calculated by using 2^-ΔΔct^ and normalized relative to GADPH.

### Western blot analysis

Cells were lysed with RIPA buffer (Beyotime, Shanghai, China) containing 1% PMSF (Beyotime). Total proteins were separated using 10% SDS-PAGE (Beyotime) and transferred to polyvinylidene fluoride (PVDF) membranes (Millipore, Billerica, MA, USA). The membrane was blocked with 5% skim milk and then incubated with primary antibody overnight at 4 °C. The bands were incubated with the HRP-conjugated secondary antibody (Sangon Biotech Co., Ltd) at room temperature for 2 h. Target proteins were visualized using an enhanced chemiluminescence kit (New Cell & Molecular Biotech Co., Ltd, Suzhou, Jiangsu, China). In this study, TLR4(Abcam, Cambridge, UK, ab13556), MyD88 (Abcam, ab133739), PD-L1 (Abcam, ab228415), p-AKT (Cell Signaling Technology, Massachusetts, USA, 4060), AKT (Cell Signaling Technology, 9272), p-Erk (Cell Signaling Technology, 4370), Erk (Cell Signaling Technology, 4695), p-Iκκα/β (Cell Signaling Technology, 2697), Iκκα (Cell Signaling Technology, 11930), Iκκβ (Cell Signaling Technology, 8943), p-NF-κB (Cell Signaling Technology, 3033), P65 (Cell Signaling Technology, 8242), p-IκBα (Cell Signaling Technology, 2859), IκBα (Cell Signaling Technology, 4814), GAPDH (Cell Signaling Technology, 5174) were used. Then expression of proteins was detected by Tanon 5200 Image System (Tanon, Shanghai, China). The relative protein expression was presented as the optical density ratio of target protein to GAPDH or Histone H3 calculated by ImageJ (National Institutes of Health, Bethesda, MD, USA).

### Immunohistochemistry, immunofluorescence, and tunnel staining

4% paraformaldehyde fixed, paraffin-embedded tissue slices were deparaffinized and rehydrated through xylene and graded alcohol series. The slices were then microwaved in citrate antigen retrieval solution (Maxim biotechnology Co., Ltd, Fuzhou, Fujian) and cooled down to room temperature. Slices were rinsed by TBS and treated with 3% H_2_O_2_ (Boster Biological Technology Co., Ltd, Wuhan, China) to quenching endogenous peroxidase. Thereafter, slices were blocked by 5% goat serum for 1 h to block nonspecific binding sites, followed by incubation with LPS(Hycult Biotech, HM6011), PD-L1 (proteintech, 66248-1-Ig), CD3 (Abcam, ab16669), CD8 (Abcam, ab209775), granzyme B (Abcam, ab255598) or CK19 (Abcam, ab52625) primary antibodies overnight at 4 °C. Further, slices were washed by TBS-T for three times and incubated with anti-Rabbit secondary antibody labeled with HRP for 2 h for PD-L1, CD3, CD8, granzyme B, CK19 and for 1 h for LPS. Lastly, Sections were covered with 3, 30-diaminobenzidine (DAB) substrate (Boster Biological Technology Co., Ltd). The PD-L1 expression was judged by the intensity of IHC staining. As for tunnel staining, the rehydrated tissue slice was stained by tunnel staining kit (Beyotime) according to the manufacturer’s protocol. As for immunofluorescence, after the slices were incubated with primary antibodies overnight, they were washed with TBS-T for three times and incubated with secondary antibody labeled with Alexa Fluor 488 or Alexa Fluor 555 for an additional 2 h. After that, the slices were washed with TBS-T and stained by DAPI for 2 mins.

### T cell separation, culture and cytotoxicity assay

Peripheral blood mononuclear cells (PBMCs) were separated from healthy human donor peripheral blood by ficoll through density-gradient centrifugation. T cells were further separated from PBMCs by Pan T Cell Isolation Kit (Miltenyi Biotec) according to the manufacture’s protocol. T cells were expanded and activated by incubating with RPMI1640 containing recombinant human IL-2 (BioLegend) (10 ng/mL) in anti-CD3 antibody (BioLegend) (10 μg/mL) and anti-CD28 antibody (10 μg/mL) (BioLegend) pre-coated tissue culture plate. PANC-1 and BxPC-3 were seeded in 24-well plates and stimulated with 10 μg/mL LPS or PBS as control. After 24 h, cancer cells were washed twice with PBS, and then T cells were added into wells at a ratio of 20:1. Anti-human PD-L1 or IgG isotype (20 μg/mL) (Bio X cell) as control were used to block the cancer cell endogenous PD-L1. After co-culturing for 7 days, cells were washed with PBS for three times, followed by 4% paraformaldehyde fixing for 20 min and stained by crystal violet solution. The ratio of colony number in each well to colony number in T cell+ IgG well was calculated as relative colony number.

### Transfection

Cells were seeded in a six-well plate at 1 × 10^6^ cells/well before transfection and reached to approximately 90% confluence. After then, they were transfected with TLR4 sh-plasmid (shRNA target sequence, sh1(5’-GCTTCATAAGCTGACTTTAAG-3’), sh2(5’-GCAGTCGTGCTGGTATCATCT-3’), sh3(5’-GCCTTTGTTATCTACTCAAGC-3’)), MyD88-sh plasmid (shRNA target logical sequence, sh1(5’-GCATATCTTTGCTCCACTTTC-3’), sh2(5’-GGACTTTGAGTACTTGGAGAT-3’), sh3(5’-GAGGGCCTATTTCCCATGATT-3’)) and control plasmid (shRNA target sequence, 5’-TTCTCCGAACGTGTCACGT-3’) according to lipofectamine 3000’s manual (Sigma-Aldrich). After incubation for 8 h at 37 °C, the supernatant was substituted by fresh medium. Panc02 cell lines were transfected with GFP-plasmid and GFP^+^ cells were sorted by FACS Cell Sorter (AriaII) after 5 days transfection.

### Cell immunofluorescence staining

Pancreatic cancer cells were treated with LPS or same volume PBS as a control for 1 h and fixed with 4% paraformaldehyde (Sangon Biotech Co., Ltd) for 20 min. Cells were incubated with 0.1% Triton X-100 (Sangon Biotech Co., Ltd) to permeabilize the membrane for 20 min and 5% goat serum solution for 1 h to block potential non-specific antibody binding. After that, cells were incubated with NF-κB P65 primary antibody (Cell Signaling Technology, 3 Trask Lane, Danvers, MA 01923) solution overnight at 4 °C. Further, cells were washed with PBS and incubated with Alexa 488-conjugated anti-Rabbit secondary antibody (Beyotime) for 1 h. After that, cells were stained by DAPI (Beyotime) for 3 min. Finally, images were acquired by using fluorescence microscope after gently washing.

### Nuclear cytoplasmic fractionation

The cell nuclear and cytoplasmic proteins were isolated according to the Nuclear and Cytoplasmic Protein Extraction Kit (Beyotime, Shanghai, China) manual. Nuclear and cytoplasmic protein were further measured by western blot and Histone-H3 or GAPDH were as used as reference, separately.

### Chromatin immunoprecipitation (CHIP) assay

5 × 10^7^ cells were crosslinked with 1% paraformaldehyde, and the crosslinking was terminated by 0.125 M glycine solution. After lysed with SDS lysis buffer containing proteinase inhibitors (Beyotime, Shanghai, China) and 1 mM PMSF (Beyotime). The cell lysates were ultra-sonicated, and the chromatin fraction were incubated with a specific P65 antibody (Abcam) or IgG as a negative control. Input, CHIP, and negative control DNA were amplified, and qRT-PCR was performed to analyzed the target DNA fragments. The specifically designed primers sequences for qRT-PCT were listed in Table S3.

### Statistical analysis

SPSS 22.0 software (IBM Corporation, NY, USA) and GraphPad Prism 6.0 (GraphPad Software, La Jolla, CA, USA) were used for statistical analysis and diagram. The correlations between TLR4, LPS, LBP, LPS/HDL, and PD-L1 were analyzed by Pearson or Spearman test based on Kolmogorov-Smirnov test for normal distribution. All the experimental results were presented as means ± standard deviation from three independent experiments. Data with two groups were analyzed by two-tailed Student’s *t*-test, and data with multiple groups were compared with a one-way analysis of variance. *P* < 0.05 was considered as statistical significance.

## Supplementary information

Figure S1

Figure S2

Figure S3

Figure S4

Figure S5

Figure S6

Figure S7

supplementary figure legends

Table S1

Table S2

Table S3

## Data Availability

All the data generated or analyzed during this study are included in this published article and its supplementary files. Further details are available from the corresponding author on reasonable request.
