# Supplementary figures and images for: Gut-derived lipopolysaccharide remodels tumoral microenvironment and synergizes with PD-L1 checkpoint blockade via TLR4/MyD88/AKT/NF-κB pathway in pancreatic cancer
Source: Cell Death Dis. 2021 Oct 30;12(11):1033. doi: 10.1038/s41419-021-04293-4 (PMC8557215; doi:10.1038/s41419-021-04293-4)

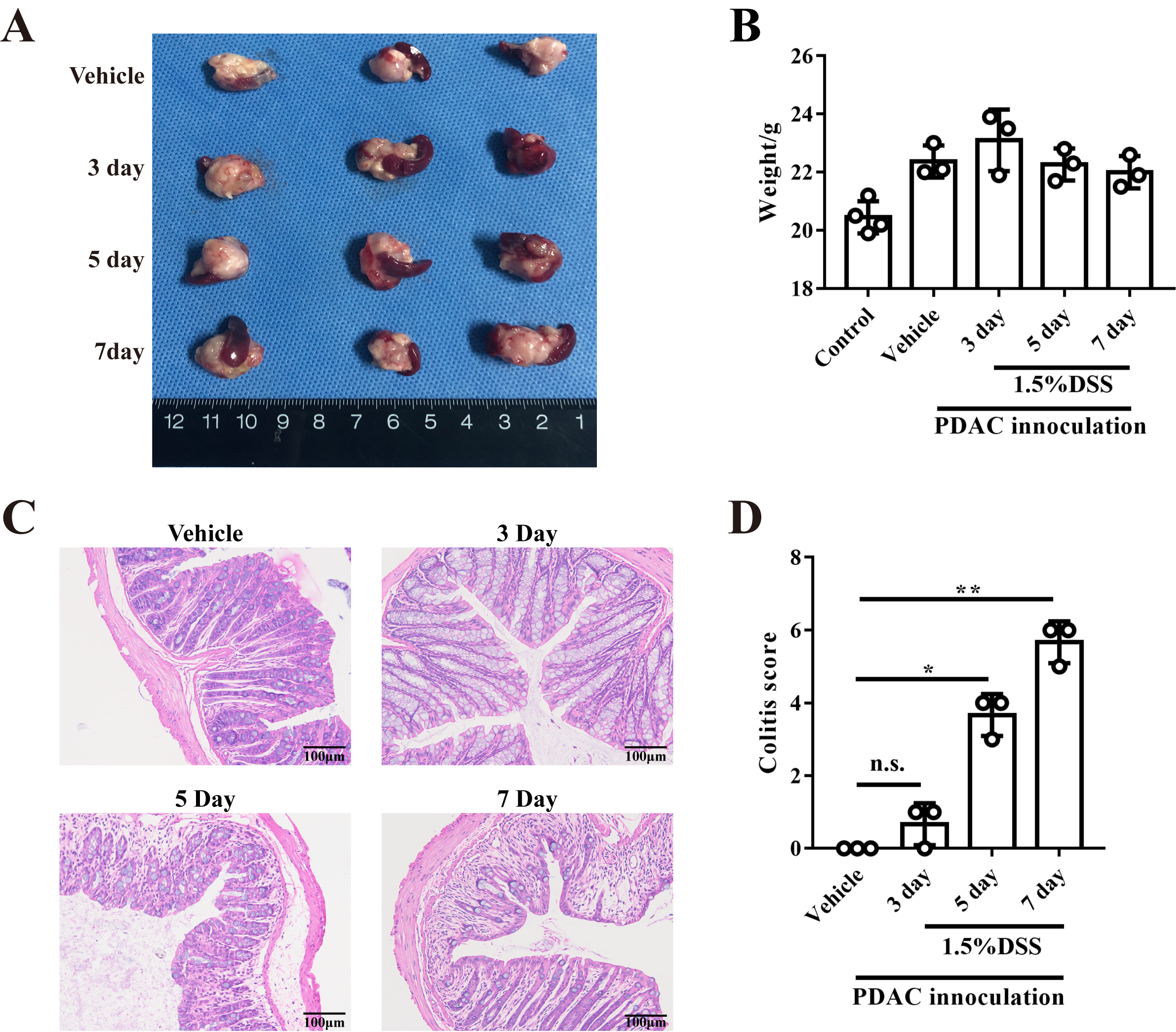

Supplement: Supplementary file 1 — Figure S1 [file 41419_2021_4293_MOESM1_ESM.tif]

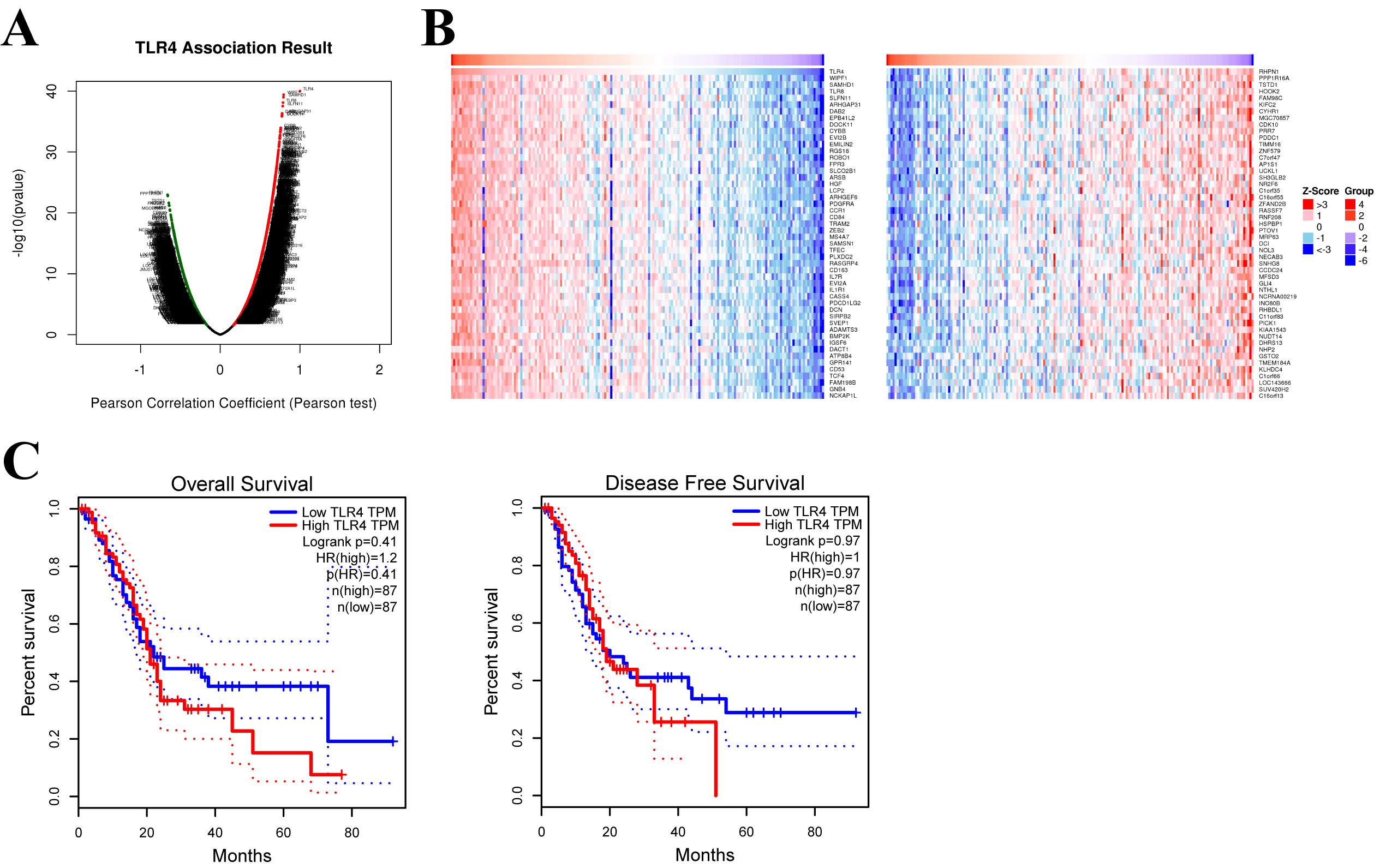

Supplement: Supplementary file 2 — Figure S2 [file 41419_2021_4293_MOESM2_ESM.tif]

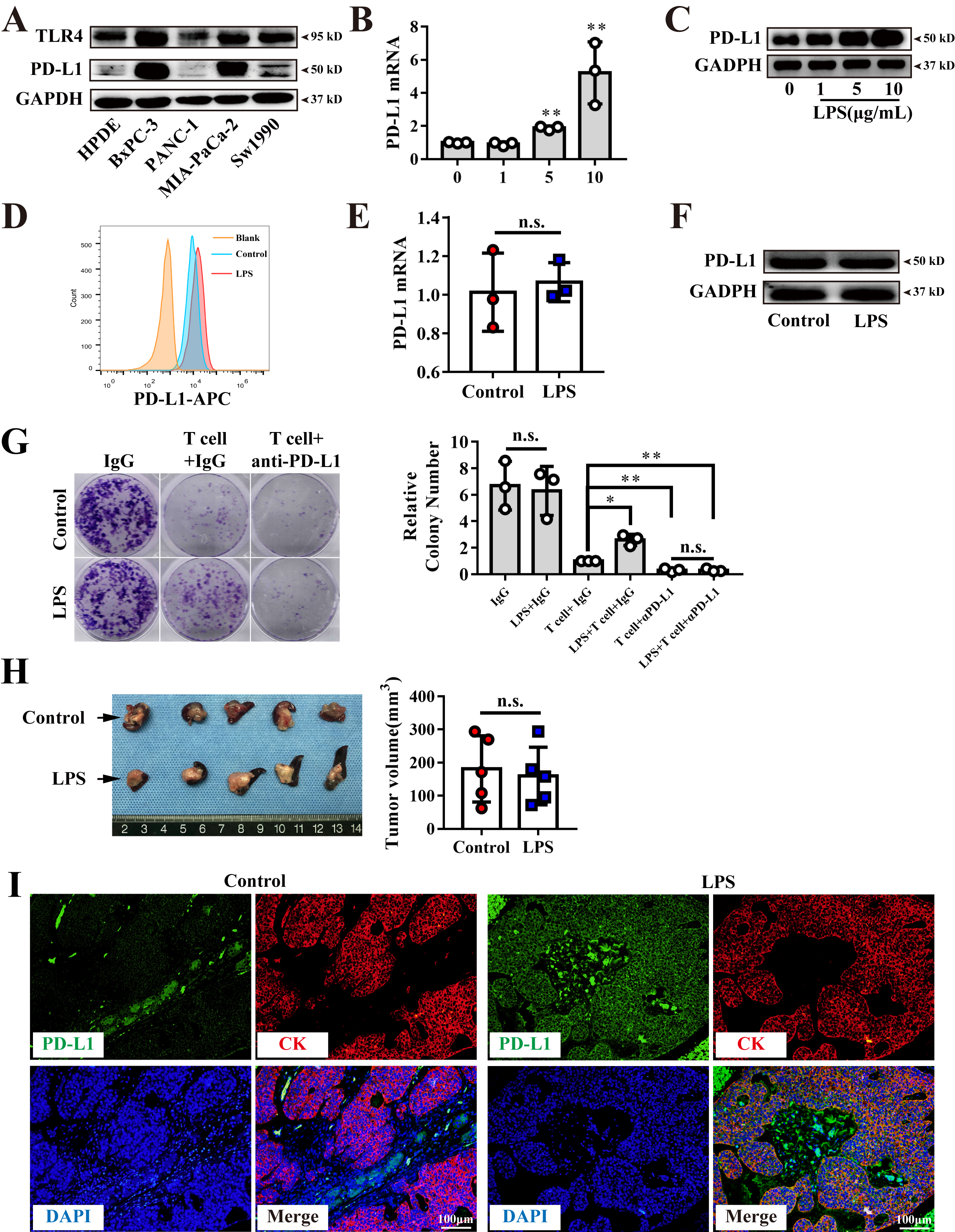

Supplement: Supplementary file 3 — Figure S3 [file 41419_2021_4293_MOESM3_ESM.tif]

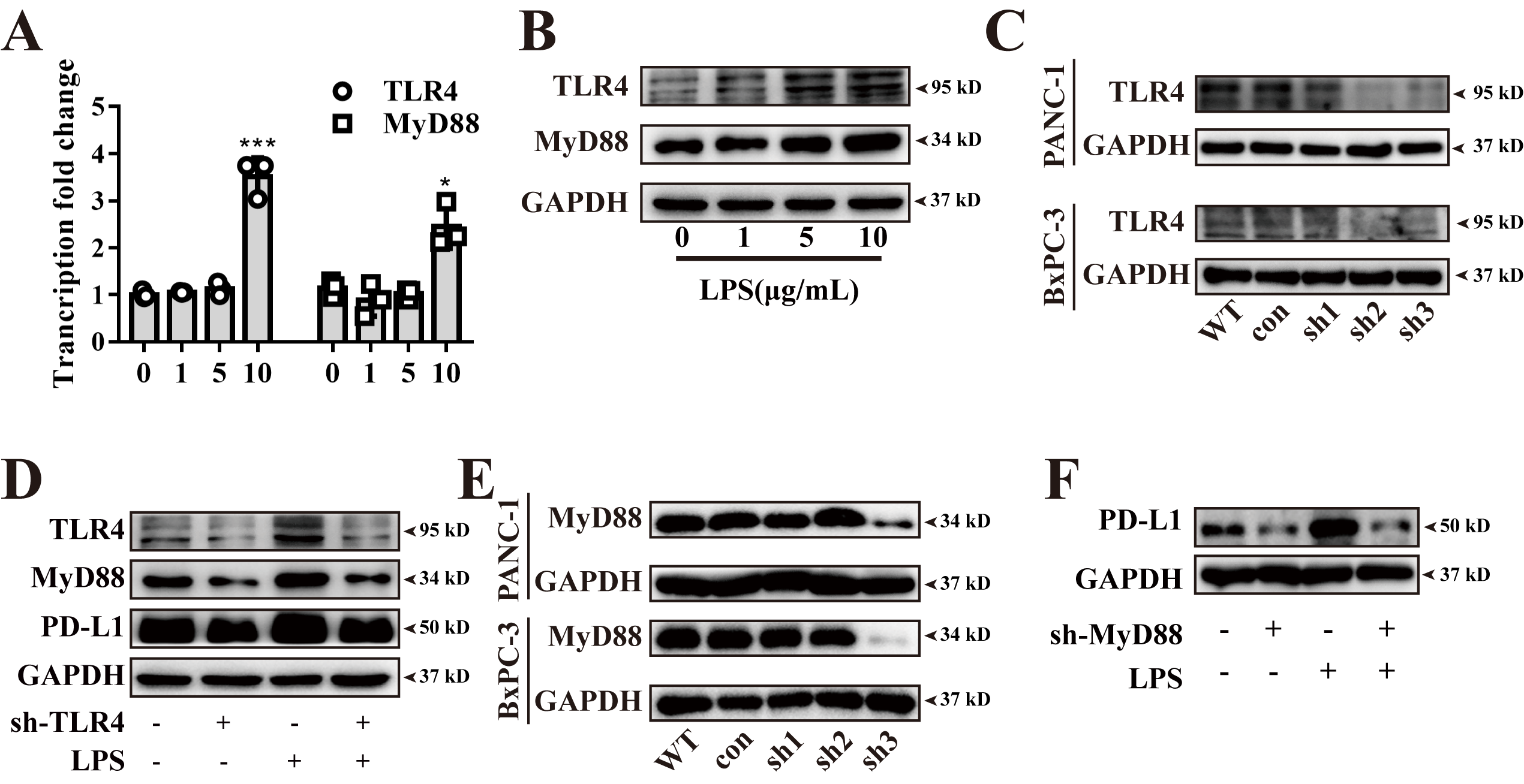

Supplement: Supplementary file 4 — Figure S4 [file 41419_2021_4293_MOESM4_ESM.tif]

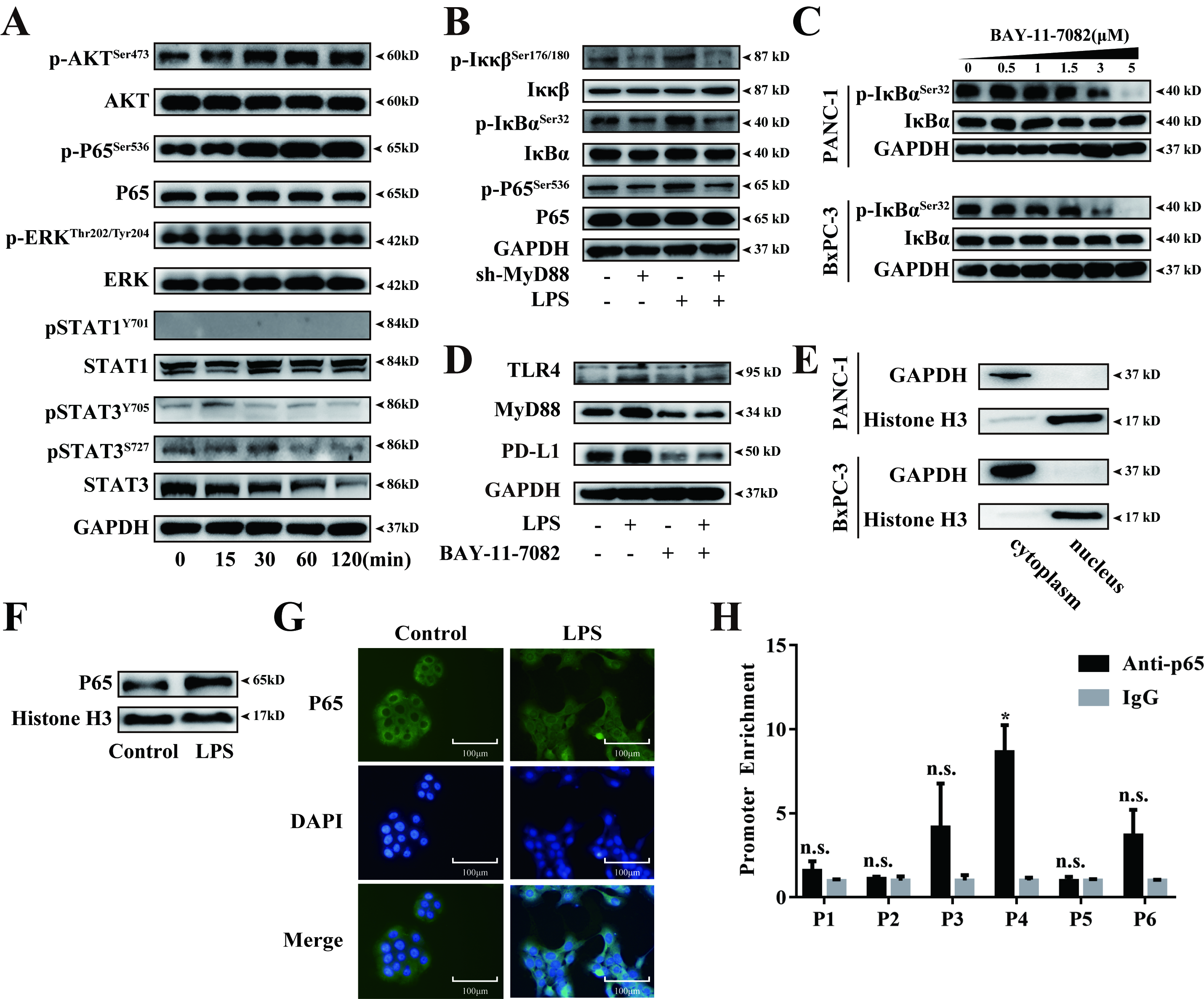

Supplement: Supplementary file 5 — Figure S5 [file 41419_2021_4293_MOESM5_ESM.tif]

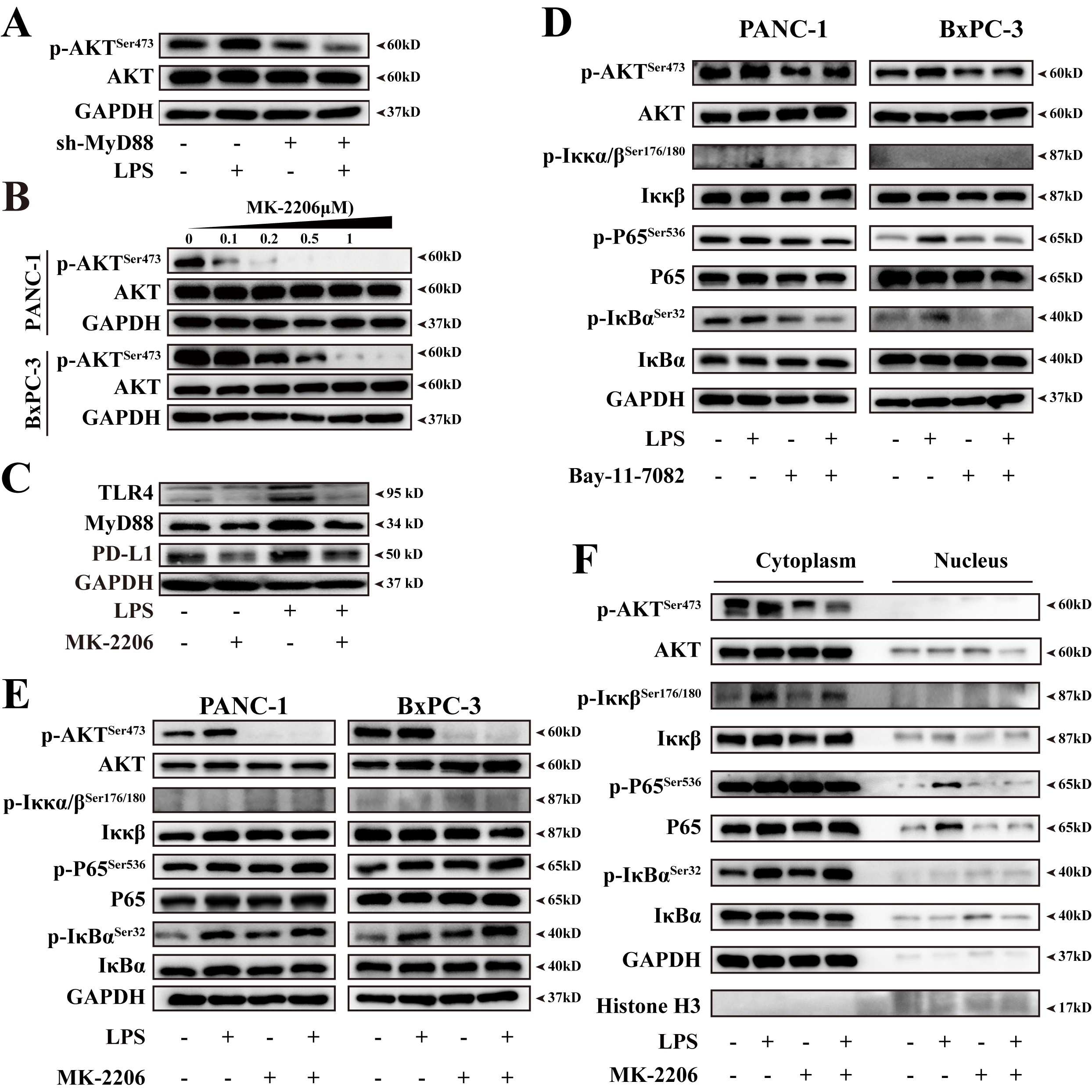

Supplement: Supplementary file 6 — Figure S6 [file 41419_2021_4293_MOESM6_ESM.tif]

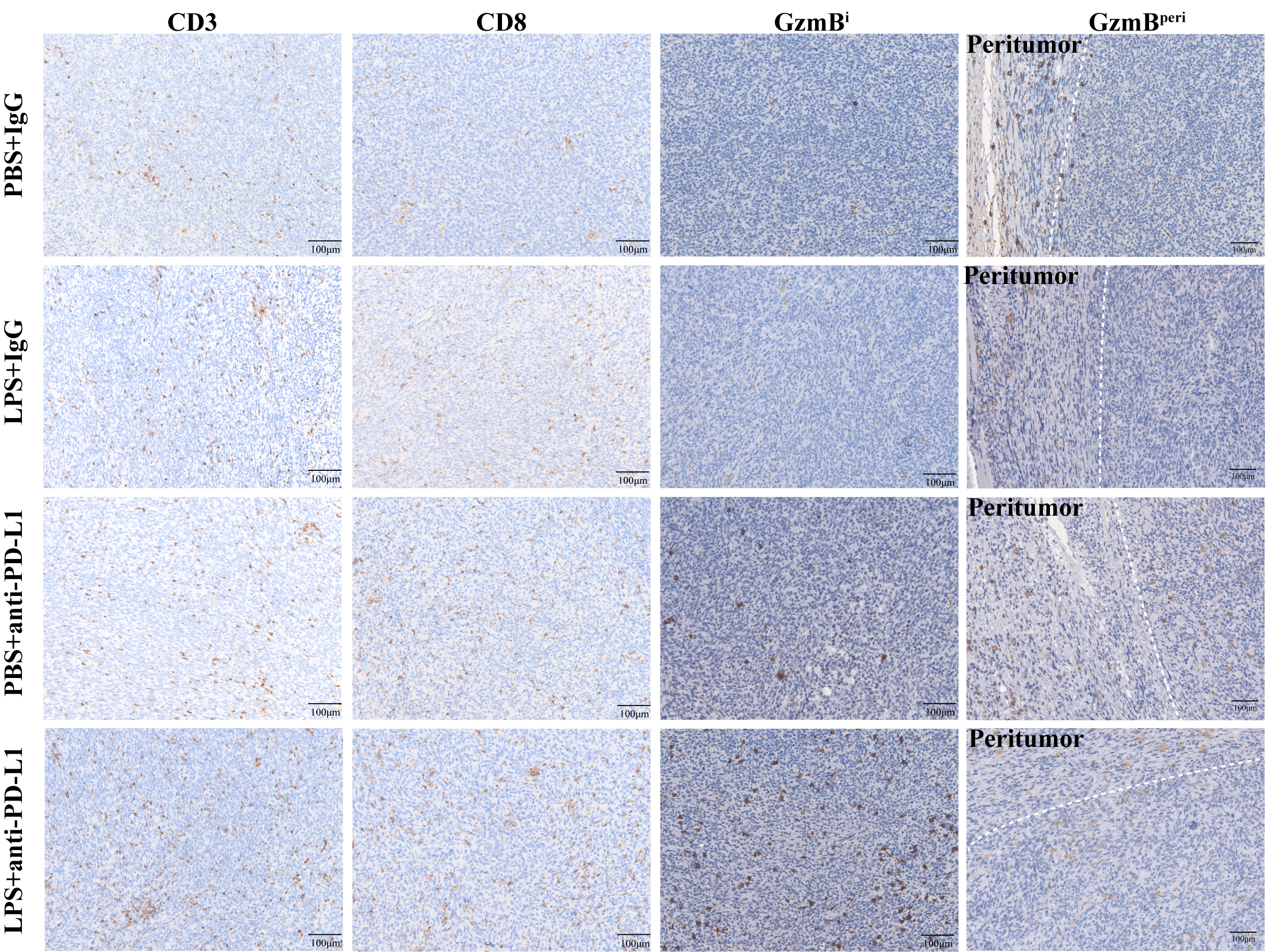

Supplement: Supplementary file 7 — Figure S7 [file 41419_2021_4293_MOESM7_ESM.tif]
