# Supplementary material for: Gut-derived lipopolysaccharide remodels tumoral microenvironment and synergizes with PD-L1 checkpoint blockade via TLR4/MyD88/AKT/NF-κB pathway in pancreatic cancer
Source: Cell Death Dis. 2021 Oct 30;12(11):1033. doi: 10.1038/s41419-021-04293-4 (PMC8557215; doi:10.1038/s41419-021-04293-4)
Supplement: Supplementary file 8 — supplementary figure legends [file 41419_2021_4293_MOESM8_ESM.docx]

**Fig. S1 DSS induced colitis in PDAC burden mouse.** **A** orthotopic tumor tissues from different DSS treated time group (n=3). **B** mice weight after DSS treatment. **C** representative images of colon after DSS treatment. **D** Histologic colitis score for mice in (**A**).

**Fig. S2** **Bioinformatic analysis showed the related gene of TLR4 and patient survival.** **A** the volcano plot generated by GEPIA showed highly correlated genes with TLR4 in PDAC. **B** Heat map showed the top 50 genes positively (left panel) and negatively (right panel) correlated with TLR4. **C** 174 PDAC patient data in TCGA database had between equally divided into TLR4^hi^ and TLR4^low^ group, both groups contained 87 cases. Disease-free survival (DFS) and overall survival (OS) were further analyzed in these cases.

**Fig. S3 TLR4 constitutive expressed in pancreatic cancer and LPS induced PD-L1 expression in BxPC-3 cell line. A** western blot measured the basic expression of TLR4 and PD-L1 in four pancreatic cancer cell lines (BxPC-3, PANC-1, MIA-PaCa-2, Sw1990) and normal pancreatic epithelium (HPDE). qRT-PCR assay (**B**), western bolt (**C**) and flowcytometry (**D**) showed that LPS induced PD-L1 expression in BxPC-3. The same treatment was executed in HPDE and PD-L1 expression were measured by qPCR assay (**E**) or Western bolt (**F**). **G** T cell cytotoxicity to tumor cells were evaluated by BxPC-3 clonal formation with or without 10ug/mL LPS pretreatment for 24 hours, PD-L1 antibody were used to neutralized cancer cell PD-L1 signaling. **H** BxPC-3 cell line derived xenograft at the end of the experiment and the tumor weight of BxPC-3 xenograft tumor (n=5). **I** PD-L1(green) and CK19 (red) co-immunofluorescence staining was analyzed for PD-L1 expression in BxPC-3 xenografts after LPS treatment.

**Fig. S4** **LPS induced TLR4 and MyD88 expression in BxPC-3 cell lines.** qRT-PCR **(A)** and western bolt **(B)** showed that LPS stimulation promoted TLR4 and MyD88 and PD-L1 expression in BxPC-3. **C** western blot analyzed TLR4 knockdown efficiency in PDAC cell lines. **D** western blot analyzed TLR4, MyD88 and PD-L1 expression after TLR4 knockdown in BxPC-3. **E** western blot showed MyD88 knockdown efficiency in PANC-1 and BxPC-3 cell lines. **F** representative western blot of PD-L1 expression after MyD88 knockdown in BxPC-3. *, ** and n.s. means statistically significant difference at p<0.05, p<0.01, p<0.001 and no significant, respectively.

**Fig. S5 NF-κB pathway promoted PD-L1 expression in LPS stimulation in BxPC-3.** **A** representative western blot of MAPK, JAK-STAT, AKT and NF-κB pathway phosphorylation after 10ug/mL LPS stimulation in BxPC-3. **B** western blot analysis of NF-κB activation in BxPC-3 after MyD88 knockdown and LPS stimulation. **C** different concentration of NF-κB inhibitor effect on the phosphorylation of IκBα, 5μM Bay-11-7082 was regarded as optimal efficiency and were used in following experiment. **D** BxPC-3 was pre-treated with bay-11-7082 and western blot showed the expression of TLR4, MyD88 and PD-L1. **E** nuclear and cytoplasmic proteins were separated and validated by western blot. **F** western blot showed the nuclear P65 level in BxPC-3 with or without LPS stimulation for 1 hour. **G** P65 immunofluorescence staining showed P65 nuclear translocation after LPS stimulation for 1 hour in BxPC-3. **H** CHIP-qPCR assay showed the binding of P65 to the promoter region of PD-L1 in BxPC-3, anti-P65 was used as experimental group and anti-rabbit IgG was used as control. * and n.s. means p<0.05, no significant, respectively.

**Fig. S6 AKT pathway participated in LPS-induced PD-L1 expression and interacted with NF-κB pathway in BxPC-3.** **A** representative western blot showed AKT pathway activation after MyD88 intervene. **B** western blot showed the efficacy of different concentration of AKT inhibitor, MK-2206, and 3μM MK-2206 had optimal efficiency in pathway inhibition. **C** representative western blot showed TLR4, MyD88 and PD-L1 expression after AKT inhibitor pretreatment in BxPC-3. Representative western blot of whole cell lysate of bay-11-7082 (**D**) and MK-2206 (**E**) pretreatment for 2 hours before LPS stimulation. **F** western blot analyzed cytoplasmic and nuclear protein in AKT and NF-κB pathway.

**Fig. S7** Representative image of immunochemistry staining of CD3, CD8, GzmB^i^ and GzmB^p^ showed the distribution of in Panc02 tumor tissue.
