# Supplementary material for: Gut-derived lipopolysaccharide remodels tumoral microenvironment and synergizes with PD-L1 checkpoint blockade via TLR4/MyD88/AKT/NF-κB pathway in pancreatic cancer
Source: Cell Death Dis. 2021 Oct 30;12(11):1033. doi: 10.1038/s41419-021-04293-4 (PMC8557215; doi:10.1038/s41419-021-04293-4)
Supplement: Supplementary file 9 — Table S1 [file 41419_2021_4293_MOESM9_ESM.doc]

**Table S1. The clinicopathological characteristics of total enrolled patients with resected PDAC.**

| **Characteristics** | **Patients** |
| --- | --- |
| **Total** | 30 |
| **Gender** |  |
| Male/Female | 18/12 |
| **Age** |  |
| < 70 / ≥ 70 | 21/9 |
| **Differentiation** |  |
| Low/Moderate/High | 16/14/0 |
| **T classification** |  |
| ≤ 4cm / > 4 cm | 23/7 |
| **N classification** |  |
| N0 / N1-2 | 13/17 |
| **TNM stage** |  |
| I / II / III | 10/14/6 |
| **CA19-9** |  |
| < 37/ ≥37 U/L | 14/16 |
| **CEA** |  |
| < 5/ ≥5 ng/mL | 23/7 |
| **TBIL** |  |
| ≤20.4 / > 20.4 mmol/L | 22/8 |
| **Glucose** |  |
| ≤5.6 / > 5.6 mmol/L | 15/15 |
| **Albumin** |  |
| < 35 / ≥35 g/L | 1/29 |
