# Supplementary material for: Gut-derived lipopolysaccharide remodels tumoral microenvironment and synergizes with PD-L1 checkpoint blockade via TLR4/MyD88/AKT/NF-κB pathway in pancreatic cancer
Source: Cell Death Dis. 2021 Oct 30;12(11):1033. doi: 10.1038/s41419-021-04293-4 (PMC8557215; doi:10.1038/s41419-021-04293-4)
Supplement: Supplementary file 10 — Table S2 [file 41419_2021_4293_MOESM10_ESM.doc]

**Table S2. Scoring systems for HAI**

| **Score** | **Colon Tissue Damage** | **Lamina Propria Inflammatory cell infiltration** |
| --- | --- | --- |
| **0** | None | Infrequent |
| **1** | Isolated focal epithelia damage | Increased, some neutrophils |
| **2** | Mucosal erosions and ulcerations | Submucosal presence of inflammatory cell clusters |
| **3** | Extensive damage deep into the bowel wall | Transmural cell infiltrations |
