# Supplementary material for: Gut-derived lipopolysaccharide remodels tumoral microenvironment and synergizes with PD-L1 checkpoint blockade via TLR4/MyD88/AKT/NF-κB pathway in pancreatic cancer
Source: Cell Death Dis. 2021 Oct 30;12(11):1033. doi: 10.1038/s41419-021-04293-4 (PMC8557215; doi:10.1038/s41419-021-04293-4)
Supplement: Supplementary file 11 — Table S3 [file 41419_2021_4293_MOESM11_ESM.doc]

**Table S3. The primer sequences used for CHIP-qPCR assay**

| Primer 1 |  |
| --- | --- |
| Forward | 5-TGTTTTTCAATCTCCGGGTA-3 |
| Reverse | 5-ATTTGCGTATTAGTAATGTGA-3 |
| Primer 2 |  |
| Forward | 5-CTCACATTACTAATACGCAAA-3 |
| Reverse | 5-TAAATGATCAATGAGGCAAAC-3 |
| Primer 3 |  |
| Forward | 5-CTGTATTGCCACATAATGTCT-3 |
| Reverse | 5-GGTCACATAGTAGAATAGAGCA-3 |
| Primer 4 |  |
| Forward | 5-CAAAGAGAACTCCATGCTCC-3 |
| Reverse | 5-ATATTGAGATAGCCCTTGCATT-3 |
| Primer 5 |  |
| Forward | 5-TTCTTAAAAGATGTAGCTCGG-3 |
| Reverse | 5-TTTAAATCGTGGATTCTGT-3 |
| Primer 6 |  |
| Forward | 5-GTCACCTTGAAGAGGCTTT-3 |
| Reverse | 5-CGCACCTTGATTTTACCTT-3 |
